# Therapeutic assessment of chloroquine–primaquine combined regimen in adult cohort of *Plasmodium vivax* malaria from a tertiary care hospital in southwestern India

**DOI:** 10.1186/s12936-015-0824-y

**Published:** 2015-08-11

**Authors:** Kumar Rishikesh, Asha Kamath, Manjunatha H Hande, Sudha Vidyasagar, Raviraja V Acharya, Vasudeva Acharya, Jayaprakash Belle, Ananthakrishna B Shastry, Kavitha Saravu

**Affiliations:** Department of Medicine, Kasturba Medical College, Manipal University, Madhav Nagar, Manipal, 576104 Karnataka India; Department of Community Medicine, Kasturba Medical College, Manipal University, Madhav Nagar, Manipal, India

**Keywords:** Malaria, *Plasmodium vivax*, Anti-malarials, Treatment failure, Chloroquine, Primaquine

## Abstract

**Background:**

Of late there have been accounts of therapeutic failure and chloroquine resistance in *Plasmodium vivax* malaria especially from Southeast Asian regions. The present study was conducted to assess the therapeutic efficacy of chloroquine–primaquine (CQ–PQ) combined regimen in a cohort of uncomplicated *P. vivax* mono-infection.

**Methods:**

A tertiary care hospital-based prospective study was conducted among adult cohort with mono-infection *P. vivax* malaria as per the World Health Organization’s protocol of in vivo assessment of anti-malarial therapeutic efficacy. Participants were treated with CQ 25 mg/kg body weight divided over 3 days and PQ 0.25 mg/kg body weight daily for 2 weeks.

**Results:**

Of a total of 125 participants recruited, 122 (97.6%) completed day 28 follow up, three (2.4%) participants were lost to follow-up. Eight patients (6.4%) were ascertained to have mixed *P. vivax* and *Plasmodium falciparum* infection by nested polymerase chain reaction test. The majority of subjects (56.8%, 71/125) became aparasitaemic on day 2 followed by 35.2% (44/125) on day 3, and 8% (10/125) on day 7, and remained so thereafter. Overall only one therapeutic failure (0.8%, 1/125) occurred on day 3 due to persistence of fever and parasitaemia.

**Conclusions:**

CQ–PQ combined regimen remains outstandingly effective for uncomplicated *P. vivax* malaria and should be retained as treatment of choice in the study region. One case of treatment failure indicates possible resistance which warrants constant vigilance and periodic surveillance.

## Background

*Plasmodium vivax* accounts for more than 50% of total malaria burden in India [1]. Chloroquine (CQ) in a dose of 25 mg/kg body weight over 3 days remains the recommended first-line treatment for all uncomplicated *P. vivax* malaria in India [2]. Additionally, for radical cure, a primaquine (PQ) dose of 0.25 mg/kg body weight for 14 days has been recommended in all non-gravid adult *P. vivax* cases with normal glucose-6-phosphate dehydrogenase (G6PD) activity [2]. Therapeutic and prophylactic failures of CQ in *P. vivax* have been observed in 23 countries across the globe, including India [3]. However, true resistance to CQ in *P. vivax* has been confirmed in only ten countries across the globe, excluding India [3].

Therapeutic assessment remains the mainstay of monitoring the efficacies of anti-malarial regimens and is recommended to be undertaken once every 2 years [1]. Surveillance of CQ–PQ combined regimen in *P. vivax* remains valid and provides useful indices where regional health policy mandates such [4]. This study was undertaken to determine the therapeutic efficacy of CQ–PQ combined regimen in adult *P. vivax* mono-infection cohort as per WHO’s protocol [4].

## Methods

### Study design and patients

A prospective cohort study was conducted among microscopically proven symptomatic mono-infection *P. vivax* patients aged ≥18 years attending Kasturba Hospital (KH), Manipal from September 2012 until October 2014. Patients were excluded if they had other febrile illnesses including mixed malaria or *P. vivax* malaria treated with artesunate combination therapy or prior anti-malarial medication outside KH before presentation or pregnancy or unwillingness to provide a written informed consent.

### Ethics statement

Ethical approval (IEC 193/2011) was obtained from the institutional ethics committee of Kasturba Medical College and Kasturba Hospital, Manipal University, Manipal. Participation in study was strictly voluntary and each participant had privilege to retract their participation any time throughout the study. Patients’ information and study data were anonymized.

### Sample size calculation

Presuming the proportion of anti-malarial treatment failure (ATF) of CQ–PQ combined regimen to be 5%, for a desired precision of 5% and confidence interval of 95%, a cohort of 73 microscopically proven *P. vivax* mono-infection cases were required [4]. Furthermore, to adjust for a 20% expected loss to follow-up and 20% expected withdrawal/protocol violation the final required sample size was determined as 122 subjects.

### Blood sample collection and processing

The date of initiation of anti-malarial medication and study enrolment were the same and was considered as day 0. Peripheral blood smear was examined on days 0, 2, 3, 7, 14, 21, and 28. Before initiation of anti-malarial medications, 1 ml venous blood sample was obtained from each participant in an ethylenediaminetetraacetic acid (EDTA) anticoagulated vacutainer and used for making peripheral smears, total leukocyte count and DNA extraction. On subsequent follow-up days capillary blood sample was obtained by finger prick on cleaned glass slides to prepare both thin and thick smears. Leishman’s stained thin and thick peripheral blood smears were examined to ascertain the *P. vivax* mono-infection and to estimate the parasite index (PI), respectively. DNA was extracted from 200 µL of EDTA anticoagulated blood using QIAamp DNA Blood Mini Kit as per the manufacturer’s instructions and stored at −20°C until conduct of nested polymerase chain reaction (nPCR) test for confirmation of the mono-infection with *P. vivax* malaria.

### Variables

#### Independent variables

Axillary temperature was recorded on each respective follow-up day as mentioned above. Participants’ total leukocyte count per µL was estimated in constituent laboratory of KH, Manipal by Beckman Coulter^®^ LH 750 Haematology Analyzer and used for PI calculation. PI was determined as the absolute number of asexual and/or sexual parasites present in 1 µL of peripheral blood, as described elsewhere [5]. Peripheral blood smears were examined under Olympus CH20i microscope and a mean PI by three consecutive calculates was derived for further analysis. Relevant demographic and clinical data of participants were recorded using a customized proforma.

### Dependent variable

ATF was considered as the primary outcome. Anti-malarial therapeutic responses were classified as per WHO’s recommendation for *P. vivax* [4]. However, for in-hospital patients ‘deteriorating clinical condition requiring hospitalization with presence of parasitaemia’ was not considered as ATF, owing to the study setting being a tertiary care hospital and the patients being hospitalized as per the clinicians’ judgement before the diagnosis of malaria and initiation of anti-malarial treatments.

### Confirmation of *Plasmodium vivax* mono-infection by nPCR test

Small sub-unit ribosomal RNA was amplified using genus and species-specific oligonucleotide primers separately for only *P. vivax* and *P. falciparum,* as described by Snounou et al. [6], with modifications in the amplification conditions of the second reaction. All reactions were carried out in a final volume of 20 µL containing 2 mM MgCl_2_, 50 mM KCl, 10 mM Tris–HCl pH 8.3, 125 µM of each dNTPs, 250 nM of each oligonucleotide primers, 0.5 units of Taq DNA polymerase (Sigma-Aldrich) and 1 µL of template DNA. Product of the first amplification reaction was further amplified in second reaction as step 1: 94°C for 5 min; step 2: 94°C for 30 s; step 3: 55°C for 30 s; step 4: 72°C for 1 min; steps 2–4 for 10 cycles, then step 6: 94°C for 10 s; step 7: 60°C for 10 s; step 8: 72°C for 1 min; steps 6–8 for 20 cycles and then final extension at 72°C for 5 min followed by termination temperature at 4°C. Reference samples of mono-infection *P. vivax* and *P. falciparum* were procured from the National Institute of Malaria Research (ICMR), Sector 8, Dwarka, Delhi-110077 (India). DNA extracted from the reference samples were used as positive controls in every batch of nPCR tests. While, DNA extracted from a normal healthy volunteer was used as negative control.

### Patients’ treatments

On presentation, patients were treated symptomatically as required. After confirmation of *P. vivax* malaria, specific anti-malarial medications were prescribed as per the clinicians’ judgements and the national guideline for treatment of malaria [2]. Resochin (chloroquine phosphate 250 mg) manufactured by Bayer Pharmaceuticals Private Limited Bayer House Central Avenue, Hiranandani Estate, Thane—400607, Maharashtra, India and Malirid (primaquine phosphate 7.5 mg) manufactured by IPCA Laboratories Pvt. Ltd. 123-AB, Kandivli Industrial Estate, Kandivli (West)Mumbai 400 067, Maharashtra, India was administered to the study cohort. All patients were instructed to take primaquine after having food and not to take while empty stomach. PQ was administered only after estimation of G6PD activity, i.e., immediately after completion of CQ dosage. Activity of erythrocytes’ G6PD enzyme was quantified by manual spectrophotometric kinetic ‘gold standard’ method. The enzyme activity was determined by measurement of increase in reduced form of nicotinamide adenine dinucleotide phosphate (NADPH) concentration, that strongly absorbs UV light (340 nm). Mild to moderate G6PD deficient patients’ were treated with 0.75 mg/kg stat dose of PQ follow by weekly once same dose for subsequent 7 weeks. Patients’ adherence to anti-malarial regimens was ascertained by direct observation for all inpatients during their hospital stay and for outpatients by passive reminder (group messages over mobile phones) and by documenting the emptied stripes of medicines on each follow-up day.

### Statistical analyses

Gaussian distribution of continuous variables was determined by Kolmogorov–Smirnov test. Data having normal distribution were summarized as mean with standard deviation and compared by independent t test. Skewed data were summarized as median with interquartile range and compared by Mann–Whitney U test. Categorical variables were summarized as frequency with proportion and compared by either χ^2^ test or Fischer’s exact test. All tests of significance were two-tailed with a p value <0.05 indicating statistical significance. Parasite index was summarized as geometric mean with 95% confidence interval of geometric mean. The probability of therapeutic failure was assessed by life table analysis. Data analysis was done using Statistical Package for the Social Sciences version 15.0 (SPSS, South Asia, Bangalore, India).

## Results

### Patients’ clinical-demographic profile

A total of 125 participants sustaining the study selection criteria were enrolled. Pertinent clinical and demographic characteristics of the study cohort have been summarized in Table 1. Significantly (p < 0.05) higher axillary temperature at presentation and longer defervescence time was noted among hospitalized than ambulatory care participants. Mean duration of hospitalization of inpatients was 4.3 ± 1.5 days.

**Table 1:** Clinical-demographic profile of study participants (N = 125) with *P. vivax* monoinfection from southwestern India treated with chloroquine (25 mg/kg over 3 days) followed by primaquine (0.25 mg/kg daily for 14 days)

Settings	Outpatient (N = 41)		Inpatient (N = 84)		p value^$^
Variables	Mean ± SD/median (IQR)	Range	Mean ± SD/median (IQR)	Range	
Age in years	33.6 ± 13.2	18–76	35.3 ± 13.3	18–73	0.49
Duration of fever in days	3 (2, 4)	1–30	4 (3, 6)	1–60	0.14
Axillary temperature at presentation (^o^C)	99.2 ± 1.0	98.6–102	100.3 ± 1.7	98.6–104	<*0.001*
Defervescence time in hours	8 (0, 17)	0–48	12 (4.5, 33.5)	0–148	*0.001*
Parasite index on day ‘0’ (parasites/µL)	1,164 (803–1,686)^a^	41–11,640	1,255 (932–1,690)^a^	64–21,700	0.17

Settings	Outpatient (N = 41)		Inpatient (N = 84)		p value^$^
Variables	Frequency (%)		Frequency (%)		
Gender (males)	39 (95.1)		71 (84.5)		0.14
Empiric antibiotics	0		1 (1.2)		1.00
Treatment failure	0		1 (1.2)		1.00
nPCR proven mixed malaria	3 (7.3)		5 (6)		0.72

^a^Geometric mean with 95% confidence interval of geometric mean.

^$^p value obtained by either independent t test or Mann–Whitney U test or Chi square test as applicable, p values <0.05 are shown in italic face.

### Patients’ treatments

Chloroquine (25 mg/kg body weight over 3 days) and primaquine (0.25 mg/kg body weight daily for 2 weeks) standard anti-malarial regimen for *P. vivax* was given to all subjects except one participant whose G6PD was low (5.5 U/gm Hb), thus was given a 45 mg stat PQ dose followed by weekly dose of 45 mg PQ for next 7 weeks. Mean G6PD activity in study cohort was determined as 15.2 ± 4.1 U/gm Hb and ranged from 5.5 to 27.2 U/gm Hb. A complete adherence for the prescribed CQ–PQ regimens was noted among study cohort. Empiric antibiotic (ceftriaxone) was administered to one (1.2%) inpatient.

### Study outcomes

**Fig. 1:**
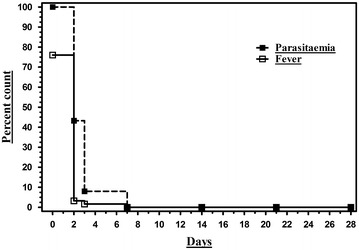
Pattern of parasitaemia and fever clearance in cohort of *P. vivax* monoinfection patients from southwestern India treated with chloroquine (25 mg/kg over 3 days) followed by primaquine (0.25 mg/kg daily for 14 days).

A total of 122 (97.6%) participants completed day 28 follow-up. Three participants (2.4%) were lost to follow up after day 3. One of them remained parasitaemic on day 3 but had become afebrile within 24 h of hospitalization. Another two participants had become aparasitaemic by day 3 and their defervescence time was less than 24 h. More than half of the subjects (56.8%, 71/125) became aparasitaemic on day 2 followed by 35.2% (44/125) on day 3; and 8% (10/125) on day 7 and remained so thereafter to day 28 (Fig. 1). Despite having history of fever within preceding 3 days, 24% (30/125) subjects were afebrile at presentation and remained so throughout study duration. Among febrile population, time to defervescence by day 2 was noted in 95.8% (91/95), by day 3 in 97.9% and by day 7 all participants had become afebrile and remained so throughout the study (Fig. 1). Overall only one ATF (0.8%, 1/125) occurred on day 3 (persistence of fever and parasitaemia, 2,200 parasite/µL on day 0 to 9,652 parasite/µL on day 2 to 7,012 parasite/µL on day 3). After this time point, attending physician changed the anti-malarial medication of that patient from CQ to artemether–lumefantrine (480–2,880 mg) combination and PQ standard regimen. Subsequently, on day 7 the patient was found to become aparasitaemic and afebrile. This one case of ATF was indeed an early treatment failure (ETF), whereas all other participants did have adequate clinical and parasitological response (ACPR) [4]. Due to only one valid survival function, i.e., therapeutic failure outcome, Kaplan–Meier plot could not be generated. The cumulative incidence of therapeutic failure by day 28 was only 1% (Table 2). No mortality occurred till 28 day follow up in the study cohort.

**Table 2:** Life table showing estimates of risk of therapeutic failure during 28 days follow-up in cohort of *P. vivax* monoinfection patients from southwestern India treated with chloroquine (25 mg/kg over 3 days) followed by primaquine (0.25 mg/kg daily for 14 days)

Days	Subjects at risk	Lost to follow up	Therapeutic failure	IR	CITF
0	125	0	0	1.00	0
2	125	0	0	1.00	0
3	125	0	1	0.99	0.01
7	124	3	0	0.99	0.01
14	121	0	0	0.99	0.01
21	121	0	0	0.99	0.01
28	121	0	0	0.99	0.01

*IR* interval risk, *CITF* cumulative incidence of therapeutic failure.

### Nested PCR results

Nested PCR test confirmed a total of 116 (93.6%) participants to have *P. vivax* mono-infection. However, the test revealed mixed (*P. vivax* and *P. falciparum*) infections among total of eight (6.4%) subjects. The DNA sample of one participant (0.8%) could not be amplified. Notably, the one subject with therapeutic failure had PCR proven *P. vivax* mono-infection.

## Discussion

Prime index of suspicion of emergence/existence of resistance to anti-malarial drugs surfaces during therapeutic drug trials or surveillances. Present study was undertaken to determine the therapeutic efficacy of CQ–PQ combined regimen in *P. vivax* malaria. The KH, Manipal caters for over 200 malaria patients annually from southwestern India comprising Goa, coastal Karnataka and Kerala. Notably, *P. vivax* infection constitutes more than 55% of total annual malaria incidence in KH, Manipal. Male preponderance observed in the current study is a common finding and has also been reported from the same hospital [7–9]. Significantly, (p < 0.05) high axillary temperature at presentation and longer time to defervescence among inpatients than outpatients is a reflection of more sick/febrile patients receiving in hospital care. Notably, about 99% therapeutic efficacy of CQ–PQ combined regimen as observed in the present study was reported previously from the same hospital during the years 2007–2009 [10]. However, the previous study did lack the confirmation of *P. vivax* mono-infection by PCR test and also did not have rigorous 28 days follow-up, as in the current study. Furthermore, similar therapeutic efficacy until day 28 has also been reported from other parts of India viz. 100% (41/41) from Mangalore [11], 98.1% (105/107) from Chennai [12] and 96.9% (63/65) from Gujarat [13] for CQ only and 100% (103/103) from Kolkata [14] for CQ–PQ combined regimen during the years 2003–2012. Notably, CQ–PQ combined regimen is known to have superior efficacy than CQ alone for preventing *P. vivax* recurrences [14–16]. Nonetheless, of the published literature from India, only a case report dating back over two decades confirms CQ failure in *P. vivax* despite having adequate plasma concentration of CQ [17].

The hub of global CQ resistance in *P. vivax* is in Indonesia [15, 18, 19]. Probable CQ-resistant *P. vivax* in India has been documented from north, northeast and western parts, whereas CQ remains sensitive throughout southern India [15]. Importantly, surging CQ resistance in *P. vivax* across eastern neighbouring countries of India, including Myanmar [20], Thailand [21] and Vietnam [22] is a matter of concern and this urges an immediate implementation of effective preventive measures to check the invasion of any resistant *P. vivax* strain from those countries along with travellers and/or migrants.

### Strengths and limitations

The present study poses considerable strength over studies which did not have PCR-proven mono-infection *P. vivax* cases. Statistically, valid sample size, robust analysis and standard methodology render the study outcomes legitimately comparable with parallel series across the globe. Outstanding therapeutic efficacy of CQ–PQ combined regimen for *P. vivax* malaria observed in the current study is augmenting evidence to support the continuation of the existing national anti-malarial drug policy for *P. vivax* malaria in India. The probably resistant case of one therapeutic failure could not be ascertained as truly resistant *P. vivax* strain. Arguably, persistence of fever and increasing parasitaemia from 2,200 parasite/µL on day 0 to 9,652 parasite/µL on day 2 and residual 7,012 parasite/µL on day 3 despite completion of CQ dosage features a true resistant *P. vivax* strain. However, this argument could possibly have been true only if CQ and desethylchloroquine concentration in participant’s blood [16, 23] was estimated on the day of therapeutic failure and/or analysis of molecular markers for CQ resistance [14, 24, 25] was done.

The nPCR test proved an insubstantial proportion of participants to have mixed malaria infection. However those entities of mixed malaria seemed inconsequential clinically as nPCR proven mixed malaria did not affect either the nature of illness or treatment/outcome and conclusions of the study. Notably, this inference of clinical inconsequentiality of merely nPCR-proven mixed malaria must be methodically evaluated further as it is beyond the scope of the current study. Lost to follow-up ensued in only three patients but seemingly they all had ACPR until their day 3 follow-up. However, we cannot rule out the possibility of treatment failure beyond day 3. Remarkably, this study did not assess the efficacy of CQ alone as per the WHO protocol [4], rather a permitted deviation from it i.e., assessment of CQ–PQ combined regimen as it was suitable in the respective study setting. Therefore, the findings of this study can only be applicable to population exempting any contraindications for PQ administration. Similar studies in future should withhold PQ administration till day 28 as recommended primarily by the WHO [4].

## Conclusions

Therapeutic efficacy of CQ–PQ combined regimen in uncomplicated *P. vivax* malaria remains intact in the study region and thus must be continued as the treatment of choice for uncomplicated *P. vivax* malaria. Periodic surveillances must be conducted to remain vigilant for taking immediate measures to prevent the emergence and spread of anti-malarial resistance in *P. vivax*.
