# Erratum to: Therapeutic assessment of chloroquine–primaquine combined regimen in adult cohort of *Plasmodium vivax* malaria from a tertiary care hospital in southwestern India

**DOI:** 10.1186/s12936-015-1055-y

**Published:** 2016-02-03

**Authors:** Kumar Rishikesh, Asha Kamath, Manjunatha H. Hande, Sudha Vidyasagar, Raviraja V. Acharya, Vasudeva Acharya, Jayaprakash Belle, Ananthakrishna B. Shastry, Kavitha Saravu

**Affiliations:** Department of Medicine, Kasturba Medical College, Manipal University, Madhav Nagar, Manipal, 576104 Karnataka India; Department of Community Medicine, Kasturba Medical College, Manipal University, Madhav Nagar, Manipal, India

## Erratum to: Malar J (2015) 14:310 DOI 10.1186/s12936-015-0824-y

The authors of the original article [1] have recently noticed that the published paper contained some errors.

In Table 2, ‘Life table showing estimates of risk of therapeutic failure during 28 days follow-up in cohort of *P. vivax* monoinfection patients from southwestern India treated with chloroquine (25 mg/kg over 3 days) followed by primaquine (0.25 mg/kg daily for 14 days)’, the column “IR (interval risk)” should have read “CSP (cumulative survival probability)”. Table 2 is correctly displayed in this erratum.

**Table 2:** Life table showing estimates of risk of therapeutic failure during 28 days follow-up in cohort of *P. vivax* monoinfection patients from southwestern India treated with chloroquine (25 mg/kg over 3 days) followed by primaquine (0.25 mg/kg daily for 14 days)

Days	Subjects at risk	Lost to follow up	Therapeutic failure	CSP	CITF
0	125	0	0	1.00	0
2	125	0	0	1.00	0
3	125	0	1	0.99	0.01
7	124	3	0	0.99	0.01
14	121	0	0	0.99	0.01
21	121	0	0	0.99	0.01
28	121	0	0	0.99	0.01

*CSP* cumulative survival probability, *CITF* cumulative incidence of therapeutic failure

Further, in the first paragraph of the ‘Discussion’ section, another case report should have been mentioned in the final sentence, to read as follows: “Nonetheless, of the published literature from India, only two case reports dating back over two decades confirms CQ failure in P. vivax despite having adequate plasma concentration of CQ [17,18].”

A new Reference 18 [2] should have been included and the subsequent references renumbered to 19–26.
